# Common coronary artery occlusions in patients with myocardial infarction

**DOI:** 10.11604/pamj.2022.42.254.34762

**Published:** 2022-08-05

**Authors:** Itedal Abdelraheem Mohamed Ahmed, Nagla Hussein Mohamed Khalid, Ahmed Elsir Mokhtar Abd-Elmagid, Mawahib Ahmed Mohammed Abdullah, Amna Mohammed Idris Musa, Nisreen Oudah AL-Qarni

**Affiliations:** 1 Faculty of Medicine, Department of Anatomy, Najran University, Najran, Kingdom of Saudi Arabia,; 2 Faculty of Applied Medical Sciences, Department of Diagnostic Radiology, Najran University, Najran, Kingdom of Saudi Arabia,; 3 College of Nursing, Department of Medical Surgical Nursing, Najran University, Najran, Kingdom of Saudi Arabia,; 4 College of Nursing, Critical Care Nursing, Najran University, Najran, Kingdom of Saudi Arabia,; 5 Faculty of Medicine, Najran University, Najran, Kingdom of Saudi Arabia

**Keywords:** Coronary artery disease, infarction, coronary and branch occlusion, coronary pattern, acute coronary syndrome

## Abstract

**Introduction:**

coronary artery disease (CAD) is a significant cardiovascular disease (CVD) that affects people worldwide. This study aimed to determine the main occluded coronary arteries in patients with myocardial infarction in Najran, Kingdom of Saudi Arabia (KSA).

**Methods:**

a retrospective cross-sectional study conducted between March 2020 and March 2021 and involving 661 myocardial infarction patients recruited from two hospitals (King Khalid Hospital and Prince Sultan Centre for Healthcare in Najran) used sampling for enrolled patients. Patients over the age of 15 years, current residents of KSA, and diagnosed with coronary artery occlusion based on at least one identifiable coronary lesion on a coronary angiography were considered eligible. We created generalized linear mixed models to investigate patients´ clinical and coronary angiographic features and identify statistically relevant components.

**Results:**

there were 661 CAD cases in this study: 548 (82.9%) males and 113 (17.1%) females, with a mean and standard deviation (SD) age of 4.03 ± 1.370 years. Ages of the 661 participants ranged from 15 to 85, who had been diagnosed with myocardial infarction were evaluated. It was found that most of the patients were in the 55-64 age range. The majority of cases (366 (55.4%) had ST-segment elevation myocardial infarction (STEMI), 187 (28.3%) had non-ST-segment elevation (NSTEMI), 101 (15.3%) had acute coronary syndrome-non-ST-segment elevation (ACS-NSTEMI), and 7 (1.1%) had acute coronary syndrome-ST-segment elevation (ACS-STEMI).

**Conclusion:**

the left anterior descending artery (LAD) is the commonest lesion found in both ST-segment elevation and non-ST-segment elevation myocardial infarction patients.

## Introduction

Coronary artery disease (CAD) is a significant cardiovascular disease (CVD) that affects a considerable portion of the global population. Ischemic heart disease (IHD) affects approximately 126 million people worldwide (1,655 per 100,000), or 1.72% of the world's population [1]. Myocardial infarction (also known as a heart attack) is the permanent death (necrosis) of the heart muscle caused by a shortage of oxygen (ischemia). Cigarette smoking, diabetes, obesity, hypercholesterolemia, and hypertension with stress play a major role in the increased incidence of myocardial infarction.

The main coronary arteries, which carry oxygen and nutrients to supply all parts of the heart, are normally implanted in a fatty tissue medium. The right and left coronary arteries arise from the proximal part of the ascending aorta. The right coronary artery (RCA) arises from the anterior aortic sinus and passes forward between the pulmonary trunk and the right atrium to slope in the right part of the atrioventricular groove. At the lower border of the heart, it continues along the atrioventricular groove to anastomose with the left coronary at the posterior interventricular groove. It gives off a marginal branch along the lower border of the heart and the posterior interventricular branch, which runs forward in the inferior interventricular groove, to anastomose near the apex of the heart with the corresponding branch of the left coronary artery (LCA) [2].

Specifically, the LCA supplies the left atrium, most of the left ventricle, part of the right ventricle, and the anterior two-thirds of the IV septum, including the atrioventricular (AV) bundle of conducting tissue, interventricular (IV) septal branches, and the sinoatrial (SA) node. The functions of conducting tissue are specialized for introducing impulses and directing them rapidly through the heart. They initiate the normal cardiac cycle and manage the contractions of cardiac chambers [2].

The RCA or the left circumflex artery (LCX) frequently give rise to the posterior descending artery (PDA), which supplies the posterior one-third of the interventricular septum. PDA originating from the left anterior descending artery (LAD) is a very uncommon occurrence. A unique kind of left dominant circulation has been reported, in which a large LAD continues as a PDA after wrapping around the apex in the presence of a tiny RCA. It is called hyper dominant when a large LAD continues as a PDA [3].

Ischemic heart disease (IHD) has claimed the lives of nine million people worldwide. Men have been more affected than women, with symptoms first appearing in the fourth decade of life and increasing in frequency with age. In the United States, coronary artery disease is the leading cause of death for both men and women. Every year, 790,000 Americans suffer from myocardial infarction. Myocardial infarction must be recognized and treated as soon as possible to improve patient outcomes [4]. Acute myocardial infarction was related to the topography of the infarction and the location of coronary artery occlusion (AMI) [5]. In Saudi Arabia, women have a high incidence of CVD risk factors, particularly obesity and physical inactivity [6].

Angina is a symptom that occurs as a result of the gradual accumulation of plaque in a coronary artery, leading to a restriction of sufficient blood flow to the heart. Angina is classified into two types: stable and unstable. Stable angina is frequently foreseeable and associated with physical and emotional stress. Stable angina symptoms do not cause permanent injury to cardiac muscle [7].

The atherosclerosis is the build-up of lipid deposits, high-density lipoproteins, also known as “good” cholesterol, and low-density lipoproteins, often known as “bad” cholesterol, and other substances within and on the walls of the arteries. Pathophysiological investigations have shown the molecular and cellular interactions in atherogenesis. In recent decades, basic research has concentrated on the instability of atherosclerotic plaque. Most acute coronary syndromes, including ST-segment elevation and non-ST-segment elevation myocardial infarction, are assumed to be caused by plaque rupture and intracoronary thrombosis. Here is a quick overview of atherosclerotic plaque pathogenesis. The plaque might cause arteries to constrict, which can prevent blood from flowing through them. There is also the possibility that the plaque will burst, resulting in a clot of blood [8].

A myocardial infarction or heart attack occurs if the cardiac muscle dies. This is the result of a coronary artery disease (CAD) plaque rupturing. Any CAD plaque can rupture; this is not enough to cause angina. When this occurs, a blood clot develops, resulting in the complete blockage of the artery and cardiac muscle death. The symptoms of a myocardial infarction (MI) are similar to those of unstable angina, but are frequently more severe and prolonged. Symptoms alone may not distinguish between unstable angina and myocardial infarction; thus, both are considered medical emergencies, and immediate medical attention is important [9].

Atherosclerosis causes nearly all coronary artery disease, peripheral arterial disease, and strokes. Atherosclerosis is a systemic inflammatory process characterized by lipids and macrophages/lymphocytes in major artery intima. Atherosclerotic rupture causes a monocyte and macrophage inflammatory cascade, thrombus development, and platelet aggregation. As a result, oxygen transport through the coronary artery is reduced, resulting in diminished myocardial oxygenation. The inability of the mitochondria to make ATP triggers the ischemia cascade, which results in endocardial apoptosis (cell death) or myocardial infarction [10].

The majority of myocardial infarctions occur in the RCA (n = 92, 44%) and LAD (n = 81, 39%) [11]. In patients with inferior and anterior wall STEMI, ECG indications of RVMI were identified in 35 and 31 (23.8% for both), respectively. The RCA was occluded in 32 ECG-RVMI patients, while the LAD or LCX arteries were occluded in 34 ECG-RVMI patients [12]. The previous findings included chronic complete occlusion CTOs of the LAD were seen in 40 (34.18%) patients, the RCA in 61 (52.14%) patients, and obtuse marginal (OM) branch of the left circumflex artery (LCX) in 16 (13.67%) patients [13]. The previous study included 139 NSTEMI patients with a mean age of 50.47 ± 12.47 years. Males made up the bulk (70.5%), with the majority (67.6%) being under 40 years of age. The LAD artery was occluded in 68.3% of NSTEMI patients, followed by the RCA in 49.6%, the LCX artery in 40.3%, and the diagonal and obtuse marginal (OM) arteries in 50.4% [14].

Previous research suggests that nearly a quarter of NSTEMI patients have a completely occluded coronary artery, with two-thirds of occlusions already collateralized at the time of angiographic examination [15, 16]. This was more common in patients presenting with either RCA or LCX involvement [15-21], which could be explained by the lack of electrocardiography (ECG) sensitivity in detecting acute ischemia in the inferolateral and posterior walls.

A myocardial bridge is the most common congenital cardiac defect. One 60-year-old male patient underwent coronary angiography and suffered a stent fracture and coronary aneurysm after cardiac bridge stenting. A myocardial bridge was discovered in the distal LAD coronary artery during coronary angiography. In the distal left anterior descending artery, a 3.0 mm, 29 mm sirolimus eluting stent was placed [20]. A previous study found that; a total of 242 individuals were included in the study, with 68.6% having a LAD artery lesion, 51.2% having an RCA lesion, and 48.8% having an LCX artery lesion. In 57.4 % of patients, multivessel disease was discovered. In terms of coronary vessel dominance, the RCA was dominant in 57.5% of patients, whereas the left coronary artery was dominant in 32.6 % [21].

Obstructive coronary lesions (50% constriction) were more common in the LAD artery (36-38%), and similar in the LCX and RCA (27-29%) [22]. The most prevalent cardiovascular lesions were LCX artery lesions (85.3%), followed by LAD artery lesions (82.4%), and right circumflex artery lesions (82.4%) after coronary angiography (74.3%). The left major coronary artery had the lowest incidence of lesions (10.3%) [23]. Coronary arteriography and left ventriculography were used to investigate the connection between collateral pathway patterns and collateral function in patients with proximal blockage of the LAD [24].

**Objective:** to determine which coronary artery is commonly occluded in patients with myocardial infarction.

## Methods

**Study type and design:** between March 2020 and March 2021, we conducted a multi-centric, retrospective, cross-sectional study to determine which coronary artery is commonly occluded in patients with myocardial infarction recruited from two hospitals (King Khalid Hospital and Prince Sultan Centre for Healthcare in Najran) in the Kingdom of Saudi Arabia (KSA) by random sample technique. We used keywords (coronary artery disease, infarction, coronary and branch occlusion, coronary pattern, and acute coronary syndrome) related to CAD epidemiology and did a thorough search of the PubMed and Google Scholar databases to find related research that we could use to plan and write this study.

**Methods of collecting data and instrumentation:** the electronic health records of patients were used to collect data. A data collection sheet with the patient's BMI, age, and gender, as well as a questionnaire about CAD symptoms and a checklist with the results of an echocardiogram and echocardiographic (ECG) assessment of the coronary arteries with myocardial infarction, were used.

**Inclusion criteria:** there were a total of 661 patients who were recruited and evaluated according to a set of severe inclusion and exclusion criteria. The following were the study's inclusion criteria: The Saudi and non-Saudi patients in the study were of all genders, both male and female, over the age of 15 years, currently residing in Saudi Arabia, presented to the King Khalid Hospital and Prince Sultan Centre for Health Care with informed consent from the legal guardian and were diagnosed with CHD based on at least one detectable coronary lesion on coronary angiography.

**Exclusion criteria:** this included patients who did not have legal guardian consent below the age of 15 years; patients diagnosed with CAD using non-invasive coronary imaging methods; patients whose coronary angiography indicated no CAD lesions; patients with incomplete or missing data points in their electronic medical records were excluded and removed; and patients with bias resulting from incomplete registration.

**Method of data analysis:** statistical analysis was performed using the statistical package for social sciences (IBM Corp, Armonk, NY, USA; SPSS Software Version 26). Frequencies and percentages were used to report categorical variables and crosstabs. We created a multinomial logistic regression model to investigate patients' clinical and coronary angiographic features that may be associated with the types of myocardial infarction. This was done so that the coronary artery could be found and the main infarcted artery could be used as a point of reference. Generalized linear mixed models were used to identify statistically relevant components. Measurement level was nominal; probability distribution multinomial and link function was generalized logit. Adjusted odds ratios (AORs) and 95% confidence intervals (CIs) were computed. A two-sided P-value of < 0.050 was considered statistically significant.

**Availability of data and materials:** if the manuscript is approved, the corresponding author will, upon reasonable request, make the datasets used and/or analyzed during the inquiry available.

**Funding:** the study was supported by Najran University's Deanship of Scientific Research, which provided funding through grant research code NU/-/MRC/10/367. The financial support organizations were not involved in the study's design, data collection, analysis and interpretation, or paper writing.

**Ethics approval and consent to participate:** the Najran University's Board of Scientific Research and Ethics Committee accepted the study protocol. The Institutional Review Board (IRB) and the King Khalid Hospital and Prince Sultan Centre for Healthcare in Najran, Saudi Arabia, have also given their approval. All participants read the purpose statement of the study, and they all gave their consent.

## Results

There were 661 cases in this study: 548 (82.9%) males and 113 (17.1%) females with coronary artery disease (CAD), with a mean and standard deviation (SD) age of 4.03 ± 1.370 years. In this study, 661 participants aged 15 years and over who had been diagnosed with myocardial infarction were evaluated; most of the patients ranged in age from 55 to 64 years. The majority of cases 366 (55.4%) had ST-segment elevation myocardial infarction (STEMI), 187 (28.3%) had non-ST-segment elevation acute coronary syndrome (NSTEMI), 101 (15.3%) had acute coronary syndrome (ASC-NSTEMI), and 7 (1.1%) had ASC-STEMI. There were 329 (89.9%) male and 37 (11.1%) female patients with STEMI; 139 (74.3%) male and 48 (25.3%) female NSTEMI patients; 5 (71.4%) male and 2 (28.6%) female patients with ASC-STEMI. The male and female patients with ASC-NSTEMI numbered 75 (74.2%) and 26 (25.9%), respectively. Almost half (310 (47%)) of the cases were overweight, ACS-NS 43 (13.9%), ACS-STEMI 4 (1.3%), NSTEMI 75 (24.2%), and STEMI 188 (60.6%). COVID-19 was diagnosed in 7 (1.1%) of the cases, 5 (71.4%) of the cases were NSTEMI, and 2 (28.6%) were STEMI (Table 1).

**Table 1:** frequency of age, gender, and BMI categories in myocardial infarction patients (N=661)

Demographic data	Categories	ACS, NSTEMI	ACS, STEMI	NSTEMI	STEMI	Total
Age (years)	15-24	0	0	7	0	7
	25-34	0	0	4	11	15
	35-44	6	0	16	35	57
	45-54	12	0	44	114	170
	55-64	32	5	63	103	203
	65-74	36	0	20	58	114
	75-85	14	2	26	33	75
	> 85	1	0	7	12	20
	Total	101	7	187	366	661
Gender	Male	75	5	139	329	548
	Female	26	2	48	37	113
	Total	101	7	187	366	661
BMI	Underweight 15-19.9	2	0	5	10	17
	Normal weight 20-24.9	41	1	58	95	195
	Overweight2 5-29.9	43	4	75	188	310
	Class I obesity 30-34.9	8	2	32	52	94
	Class II obesity 35-39.9	6	0	9	18	33
	Class III obesity >40	1	0	8	3	12
	Total	101	7	187	366	661

**ACS:** Acute coronary syndrome; **NSTEMI:** Non-ST-segment elevation myocardial infarction; **STEMI: ST**-segment elevation myocardial infarction**; ACS, NSTEMI**: Acute coronary syndrome, **Non-ST**-segment elevation myocardial infarction; **ACS, STEMI**: Acute coronary syndrome; ST-segment elevation myocardial infarction; **BMI**: Body mass index

Most myocardial infarctions occurred in the left anterior descending artery (LAD); 280 (42.4%) in the right coronary artery (RCA); 177 (26.8%), in the left circumflex artery (LCX); 126 (19.1%) in the right atrial branch (RAB); 1 (0.2%) in the left atrial branch (LAB); and 1 (0.2%) in the posterior left ventricular (PLV). It can be seen that most types of myocardial infarction are STEMI, and most of these are in the LAD (147 (52.5%) out of 280). Other types are RCA 106 (59.9%) out of 177 and LCX 72 (57.1%) out of 126; it is not present in RAB and PLV. A different type of myocardial infarction (NSTEMI) occurs in LAD 82 (29.3%) out of 280, in RCA 41 (23.2 %) out of 177, and LCX 37 (29.4%) out of 126, whereas it is not present in LAB. Non-STEMI acute coronary syndrome occurs more frequently than STEMI acute coronary syndrome. It occurs in LAD 48 (17.1%), RCA 28 (15.8%), and LCD 17 (13.5%), and is not present in RAB and PLV. Common types of myocardial infarction are STEMI, NSTEMI, and acute coronary syndrome NSTEMI. LAD is affected more frequently in most types of myocardial infarction (Table 2).

**Table 2:** distribution and association pattern of arterial lesions with myocardial infarction (N = 661)

Pattern of artery occlusion		Myocardial infarction (MI)			
Common arteries and branches	N (%)	ACS,NESTMI	ACS,STEMI	NSTEMI	STEMI
Diag.	35 (5.3%)	5	1	13	16
LAB	1(0.2%)	0	0	0	1
LAD	280(42.4%)	48	3	82	147
LCA	31(4.7 %)	1	1	10	19
LCX	126(19.1 %)	17	0	37	72
PLV	1(0.2%)	0	0	1	0
RAB	1(0.2%)	0	0	1	0
RCA	177 (26.8 %)	28	2	41	106
RPD	9 (1.4 %)	2	0	2	5
Total	661	101	7	187	366

**ACS:** Acute coronary syndrome; **NSTEMI: Non-ST**-segment elevation myocardial infarction; **STEMI: ST** -segment elevation myocardial infarction; **ACS, NSTEMI**: Acute coronary syndrome, **Non-ST**-segment elevation myocardial infarction; **ACS, STEMI**: Acute coronary syndrome; ST-segment elevation myocardial infarction N (%): Number (percentage). **RCA:** Right coronary artery; **LAD**: Left anterior descending artery; **LCX**: Left circumflex artery; Diag.: Diagonal artery; LAB: Left atrial branch; **LCA:** Left coronary artery; **PLV:** Left posterior ventricular; RAB: Right atrial branch; **RPD:** Right posterior descending artery.

The standard error for the influence of the arteries is too small in the generalized linear mixed models (GLMM) for the left anterior descending (LAD), leading to an overly optimistic result (95%; P = 0.000*, CI: 2.773-4.103). RCA (95%; P = 0.000*, CI: 2.308-3.650). LCX (95%; P = 0.000*, CI: 1.962-3.317). Diagonal artery (Diag.) (95%; P = 0.000*, CI: 0.624-2.092). LCA (95%; P = 0.001*, CI: 0.493-1.980). The remaining arteries, LAB, PLV and RAB, were effectively the same (95%; P = 0.038, CI: -4.267- -0.127) (Table 3).

**Table 3:** association between myocardial infarction and coronary arterial occlusions: generalized linear mixed models (GLMM)

Statistic	Model Terms	Std. Error	Coefficient	P-value	95 % Confidence Interval(CI)	
					Lower	Upper
**Myocardial Infarction**	Diag	.3737	1.358	.000	.624	2.092
	LAB	1.0541	-2.197	.038	-4.267	-.127
	LAD	.3386	3.438	.000	2.773	4.103
	LCA	.3786	1.237	.001	.493	1.980
	LCX	.3498	2.639	.000	1.962	3.317
	PLV	1.0541	-2.197	.038	-4.267	-.127
	RAB	1.0541	-2.197	.038	-4.267	-.127
	RCA	.3417	2.979	.000	2.308	3.650
	RPD	Ref.	Ref.	Ref.	Ref.	Ref.

**RCA:** Right coronary artery; **LAD**: Left anterior descending artery; **LCX**: Left circumflex artery; **Diag**.: Diagonal artery; **LAB**: Left atrial branch; **LCA:** Left coronary artery; **PLV**: Left posterior ventricular; **RAB**: Right atrial branch; **RPD**: Right posterior descending artery.

**Figure 1:**
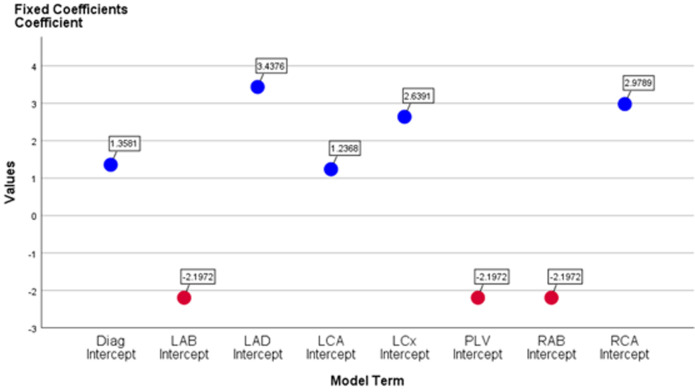
the fixed coefficients, positive and negative, in generalized linear mixed models

The coefficient estimates for all other effects are very different in effectiveness, so the interpretation of these effects is different. In particular, the effects of LAD, RCR, LCx, Diag, and LCA are statistically significant (fixed coefficients positive), whereas LAB, PLV, and RAB are insignificant (fixed coefficients negative) in the linear mixed model (Figure 1). These findings could help with ischemia and infarction diagnosis, as well as myocardial infarction patient care.

## Discussion

This cross-sectional study at King Khalid Hospital in Najran, Saudi Arabia, sought to determine which coronary artery is commonly occluded in patients with myocardial infarction. There were 661 such CAD cases in this study: 548 (82.9%) male and 113 (17.1%) female, with a mean and standard deviation (SD) age of 4.03 ± 1.370 years. In this study, 661 participants ranging in age from 15 to 85 years who had been diagnosed with myocardial infarction were evaluated. It was found that the majority of patient's were in the 55-64 age range.

In the same context and in a previous study in Mumbai, is the capital city of the Indian state of Maharashtra, males accounted for 86 (73.50%) of the 117 patients, while females accounted for 31 (26%). The participants were all over the age of 40. There were 25 patients aged 40 to 49 years (21.36%), 32 patients aged 50 to 59 years (27.35%), 43 patients aged 60 to 69 years (36.75%), and 17 patients aged > 70 years (14.52%). Over half, 63 patients (53.84%), had unstable angina/non-ST elevated myocardial infarction (NSTEMI); 32 (27.35%) had chronic stable angina (CSA); and 22 had ST elevated myocardial infarction (STEMI) (18.80%) [13]. In Croatia city in Europe; Pačarić *et al*. [24] examined 47 coronary artery bypass grafting (CABG) patients aged 30-75 years and found that almost half of the patients (49%) were over 60 years old, and 55% were males.

A previous study concluded that the right coronary artery was common and completely blocked in seven cases, Bakst *et al*. [25], in Al-Kharj city in Saudi Arabia, Aldosari *et al*. [22] found that LCX is the most frequently involved coronary artery lesion in CAD, Pierard *et al*. [5]. described how coronary angiography was used to detect the location of coronary occlusion and infarct was associated with the left main artery, the left anterior descending artery (LAD), the left circumflex, and the right coronary artery, respectively [5]. Bassan reported that roughly all individuals who survive an acute inferior wall myocardial infarction complicated by temporary heart block had significant occlusion of the LAD [26]. In a previous study in Saudi Arabia reported by Al-Ghamdi SH, Aldosari KH, and AlAjmi MM, 68.6% of 242 patients had a LAD lesion, 51.2% had an RCA lesion, and 48.8% had an LCX lesion. Five hundred and seventy-four patients had multivessel disease. About 57.5% had a dominant RCA, while 32.6% had a dominant LCA [21]. Ghanim examined 189 coronary angiograms of patients from Israel who had ST-segment elevation myocardial infarction (STEMI) with a 50% arterial narrowing and found that LAD was the most common coronary arterial lesion (36-38%), followed by RCA and LCX lesions (27-29%) [22]. The study found that LAD is the most frequently involved coronary artery lesion in CAD.

The current study's findings are similar to prior research in which other coronary lesions, such as the left anterior descending artery, have been observed more frequently, as reported by both Bassan *et al*. [26] and Ghanim *et al*. [2,1]. In addition, the findings differ from those of the earlier studies cited by both Pierard *et al*. [5] and Aldosari *et al*. [22]. In this context, most myocardial infarctions occurred in the left anterior descending coronary artery (LAD) 280 (42.4%), in the right coronary artery (RCA) 177 (26.8%), and left circumflex artery (LCX) 126 (19.1%). In contrast, they are rare in the right atrial branch (RAB) 1 (0.2%), left atrial branch (LAB) 1 (0.2%), and posterior left ventricular artery (PLV) 1 (0.2%). In addition, most types of myocardial infarction are ST-segment elevation myocardial infarction (STEMI), mainly in the left anterior descending artery (LAD), with 147 (52.5%) out of 280 cases.

The anatomical interpretation is that ST-segment elevation myocardial infarction (STEMI) caused by occlusion of the left anterior descending artery (LAD) is associated with the highest risk of adverse clinical outcomes due to the large myocardial area supplied by the LAD compared to other coronary arteries, which contributes to the domestic and global mortality rate. Furthermore, structural factors, such as a lengthy LAD looping around the LV apex, can significantly impact outcomes in individuals with anterior STEMI. These findings could help with ischemia and infarction diagnosis, as well as myocardial infarction patient care.

**Limitations:** our findings are based on data collected at the hospital. Data indicating bias due to incomplete registration was excluded because it was difficult to communicate with patient's for long-term follow-up or even with their physicians including the data of blood pressure and cholesterol.

**Recommendation:** the anatomical position of coronary atherosclerotic plaques or occlusion has played a major role in the treatment of percutaneous coronary intervention (PCI) or coronary artery bypass grafting (CABG). We recommend further studies to evaluate the relationship between patient characteristics, the location of the coronary artery occlusion in particular, and the technique of CAD-CABG or PCI treatment. Even though all patients need good medical care, there is a need to use PCI more in order to meet global recommendations and improve survival.

## Conclusion

Most myocardial infarctions occurred in the left anterior coronary artery (LAD), followed by the right coronary artery (RCA) and left circumflex artery (LCX), and were rare in the right atrial branch (RAB), left atrial branch (LAB), and posterior left ventricular (PLV). In addition, most types of myocardial infarction are ST-segment elevation myocardial infarction (STEMI), most of them in the left anterior descending artery (LAD), right coronary artery (RCA), and left circumflex artery (LCX). It is not present in the right atrial branch (RAB) or posterior left ventricular (PLV). Another type of myocardial infarction is non-ST-segment elevation myocardial infarction (NSTEMI), which occurs in the left anterior coronary artery (LAD), right coronary artery (RCA), and left circumflex artery (LCX), and is not present in the left atrial branch (LAB). There a higher occurrence of NSTEMI acute coronary syndromes than STEMI acute coronary syndromes, and it occurs in the left anterior descending artery (LAD), right coronary artery (RCA), and left circumflex artery (LCX). It is not present in RAB and PLV. Common types of myocardial infarction are STEMI, NSTEMI, and acute coronary syndromes (ACS). In most types of myocardial infarction, the LAD is more affected.

### What is known about this topic

- *Ischemic heart disease (IHD) affects approximately 126 million people worldwide (1,655 per 100,000), or 1.72% of the world's population;*
- *IHD has claimed the lives of nine million people worldwide;*
- *Most studies have reported myocardial infarctions occurring in the left anterior coronary artery (LAD), while other studies have found LCX and RCA involved globally and regionally in Saudi Arabia*.

### What this study adds

- *This is the first comprehensive study of coronary artery lesions in the Saudi population reporting on the frequency of coronary artery lesions in myocardial infarction;*
- *The left descending artery (LAD) is the most common coronary artery disease affecting all types of myocardial infarction in the Kingdom of Saudi Arabia;*
- *The coronary artery lesion pattern in the Saudi population is consistent with that of other countries, as evidenced by the contrast between the same Saudi population where LAD and LCX originated from the same LCA; left anterior descending artery (LAD) infarcts increase mortality risk.*
